# Expression and co-expression of the members of the epidermal growth factor receptor (EGFR) family in invasive breast carcinoma

**DOI:** 10.1038/sj.bjc.6602184

**Published:** 2004-10-12

**Authors:** D M Abd El-Rehim, S E Pinder, C E Paish, J A Bell, R S Rampaul, R W Blamey, J F R Robertson, R I Nicholson, I O Ellis

**Affiliations:** 1 Department of Histopathology, The Breast Unit, Nottingham City Hospital NHS Trust and University of Nottingham, Hucknall Road, Nottingham NG5 1PB, UK; 2 Department of Surgery, The Breast Unit, Nottingham City Hospital NHS Trust and University of Nottingham, Hucknall Road, Nottingham NG5 1PB, UK; 3 Tenovus Institute, Cardiff, UK

**Keywords:** EGFR family, breast cancer, immunohistochemistry, tissue microarray

## Abstract

The epidermal growth factor receptor (EGFR) family plays an important role in breast carcinogenesis. Much interest has been focused recently on its members because of their potential role as prognostic indicators in breast cancer and their involvement in cancer therapy. We have evaluated more than 1500 cases of invasive breast carcinoma immunohistochemically using tissue microarray technology to examine the expression of EGFR family receptor proteins. We have found that 20.1 and 31.8% of cases were positive for EGFR and c-erbB-2, respectively, and 45 and 45.1% of tumours overexpressed for c-erbB-3 and c-erbB-4, respectively. The expression of either EGFR or c-erbB-2 was associated with other bad prognostic features and with poor outcome. Neither c-erbB-3 nor c-erbB-4 had any association with survival. c-erbB-2 had an independent prognostic effect on overall and disease-free survival (DFS) in all cases, as well as in the subset of breast carcinoma patients with nodal metastases. Several hetero- and homodimeric combinations have been reported between the EGFR members. Those dimers can evoke diverse signal transduction pathways with variable cellular responses. We stratified cases according to their co-expression of receptors into distinct groups with different receptor-positive combinations. Patients whose tumours co-expressed c-erbB-2 and c-erbB-3, as well as those whose tumours co-expressed EGFR, c-erbB-2 and c-erbB-4 showed an unfavourable outcome compared with other groups, while combined c-erbB-3 and c-erbB-4 expression was associated with a better outcome. In cases showing expression of one family member only (homodimers), we found a significant association between c-erbB-4 homodimer-expressing tumours and better DFS. In contrast, patients with c-erbB-2 homodimer-expressing tumours had a significant poorer DFS compared with other cases. These data imply that the combined profile expression patterns of the four receptor family members together provide more accurate information on the tumour behaviour than studying the expression of each receptor individually.

The epidermal growth factor receptor (EGFR) tyrosine kinase family consists of four members: EGFR, c-erbB-2, c-erbB-3 and c-erbB-4 (Gullick and Srinivasan, 1998). All share structural homology consisting of an extracellular ligand-binding domain, a transmembrane domain and an intracytoplasmic tyrosine kinase domain (Mason and Gullick, 1995). There is a growing body of evidence that this family is involved in breast cancer development and progression (Gullick and Srinivasan, 1998). Previous studies of EGFR and c-erbB-2 reported their association with characteristics of poor prognosis. Epidermal growth factor receptor overexpression was always associated with poor outcome, manifested in a short overall survival and disease-free interval (Sainsbury *et al*, 1985; Lewis *et al*, 1990; Toi *et al*, 1994; Tsutsui *et al*, 2002), high metastatic potential (Sainsbury *et al*, 1987) and negative oestrogen receptor (ER) status (Pilichowska *et al*, 1997; Tsutsui *et al*, 2002). The same behaviour was also detected in tumours showing c-erbB-2 gene amplification and/or overexpression, which was marked in high tumour grade as well as short overall survival and disease-free interval (Winstanley *et al*, 1991; Charpin *et al*, 1997; Suo *et al*, 2002).

Relatively less information has been reported about the other two members: c-erbB-3 and c-erbB-4. Significant correlations have been found between c-erbB-3 overexpression and tumour size (Travis *et al*, 1996) and histological grade (Naidu *et al*, 1998). Studies on c-erbB-4 expression have shown an association with well-differentiated tumours (Kew *et al*, 2000; Suo *et al*, 2002) and positive ER status (Suo *et al*, 2001). Some studies have found an association with better survival (Pawlowski *et al*, 2000; Suo *et al*, 2002), while others have reported an association with poor survival (Lodge *et al*, 2003) or no association with survival (Kew *et al*, 2000).

Most of the previous research has focused on investigating the expression of individual members in relation to different clinicopathological studies. However, a few studies have shown that the co-expression of two or more members had an adverse effect on breast cancer behaviour and outcome. The best example for these findings is the reported adverse synergistic effect of EGFR and c-erbB-2 expression on both prognosis (Osaki *et al*, 1992; Toi *et al*, 1994; Suo *et al*, 2002) and metastasis (Brandt *et al*, 1999). Such studies have evoked our interest to investigate the expression of the four members together on a large number of invasive breast carcinomas with long follow-up in order to shed light on any potential prognostic implications.

## MATERIALS AND METHODS

### Patients

A consecutive series of 1944 cases of primary operable invasive breast carcinoma from patients presenting between 1986 and 1998 and entered into the Nottingham Tenovus Primary Breast Carcinoma Series were used. Data on histological grade (Elston and Ellis, 1991), histological tumour type (Ellis *et al*, 1992), vascular invasion (Pinder *et al*, 1994), tumour size, lymph node stage and Nottingham Prognostic Index (NPI) (Galea *et al*, 1992) are routinely assessed and recorded in the database. The NPI is calculated using the following equation: NPI=0.2 × tumour size (cm)+grade (1–3)+lymph node stage (1–3). Patients are assigned into three groups: good, moderate and poor. A score of ⩽3.4 indicates a good prognosis, 3.41–5.4 a moderate prognosis and >5.4 a poor prognosis.

Patient age ranged from 18 to 70 years (mean age 53, median 54 years). Mean survival was 62 months (range 1–192 months). Information on local, regional and distant recurrence and survival is maintained on a prospective basis. Patients are followed up at 3-month intervals initially, then 6 monthly and then annually, for a median period of 58 months. The disease-free interval was defined as the interval (in months) from the date of the primary surgical treatment to the first loco-regional or distant recurrence. The overall survival was taken as the time (in months) from the date of the primary surgical treatment to the time of death. Oestrogen receptor status was estimated immunohistochemically in 1805 of the tumours; 553 (30.6%) were negative for ER expression, while 1252 (69.4%) carcinomas were ER positive.

Data for histological tumour type, grade and staging are summarised in Tables 1

**Table 1:** Frequencies and percentage of histological tumour types

**Tumour type**	**No**	**%**
Invasive NST	1094	56.50
Tubular mixed	337	17.40
Medullary	46	2.40
Typical	5	
Atypical	41	
Lobular	220	11.40
Classical	141	
Alveolar	2	
Solid	6	
Tubulo-lobular	6	
Mixed	65	
Tubular	79	4.10
Mucinous	26	1.30
Invasive cribriform	10	0.50
Invasive papillary	7	0.40
Mixed NST & lobular	65	3.40
Mixed NST & special type	41	2.10
Miscellaneous other types	14	0.70
Adenoid cystic	5	
Metaplastic	3	
Spindle cell tumour	1	
Apocrine carcinoma	1	
NST with clear cell features	1	
NST with secretory features	1	
NST with spindle cell element	1	

and 2

**Table 2:** Frequencies and percentage of tumour grades, size, LN stage and distant metastases

**Grade**	**No.**	**%**
1	367	18.9
2	647	33.4
3	925	47.7
		
**LN stage**	**No.**	**%**
N0	1231	63.6
N1	549	28.4
N2	156	8.1
		
**Size**	**No.**	**%**
⩽2 cm	1220	62.9
>2–5 cm	685	35.3
>5 cm	36	1.9
		
**Distant metastases**	**No.**	**%**
M0	1701	88.8
M1	214	11.2

.

### Construction of the tissue microarray blocks

Breast cancer tissue microarrays were prepared as described previously (Kononen *et al*, 1998; Camp *et al*, 2000; Torhorst *et al*, 2001). Haematoxylin and eosin (H&E) slides were obtained from each available conventional tumour block and used as a guide for selection of the most representative areas of the tumour. Tissue microarrays were constructed by obtaining 0.6 mm diameter cylinders from the original blocks and re-embedding these cores into the recipient block. Each case was sampled twice, from the centre and the periphery of the tumour, to form an array of 100 cases per block. Histological tumour types and tumour grade are summarised in Tables 1 and 2.

### Immunohistochemistry

Immunohistochemical staining for the sections was performed according to the avidin–biotin complex method. Tissue sections of 3 *μ*m thickness were taken from tissue array blocks. The initial sections were stained with H&E to confirm the histological diagnosis. Paraffin sections were dewaxed and then rehydrated. To block the endogenous peroxidase, the rehydrated sections were treated with 0.3% hydrogen peroxide in methanol for 10 min. To unmask the antigens, sections, with the exception of those for c-erbB-2 and c-erbB-4, were microwaved in citrate buffer, pH 6 for a total 20 min. After the nonspecific staining had been blocked by normal swine serum, sections were incubated with the primary antibodies for between 50 and 60 min. The antibodies used were EGFR (clone EGFR.113, Novocastra, diluted at 1 : 10), c-erbB-2 (Dako, diluted at 1 : 250), c-erbB-3 (clone RTJ1, Novocastra, diluted at 1 : 20), c-erbB-4 (clone HFR1, Neomarkers, diluted at 6 : 4) and ER (clone 1D5, Dako, diluted at 1 : 80). Sections were incubated with the biotin-labelled secondary antibody (diluted 1 : 100) for 30 min, then in avidin–biotin complex (diluted 1 : 100) for a further 45 min. 3-3′Diaminobenzidine tetrahydrochloride was used as the chromogen.

### Controls

Positive and negative controls were included in each staining run. Positive controls were myoepithelial cells of normal duct in normal mammary gland for EGFR, renal tissue (proximal and distal tubules) for c-erbB-3 and known positive cases of breast carcinoma for c-erbB-2, c-erbB-4 and ER. Negative controls were obtained by omitting the primary antibodies.

### Immunohistochemical scoring

The modified histochemical score (H-score) (McCarty *et al*, 1985) was used as it includes a semiquantitative assessment of both the intensity of staining and the percentage of positive cells. For the intensity, a score of 0–3 corresponding to negative, weak, moderate and strong positivity was recorded. In addition, the percentage of positive cells at each intensity was estimated in %. The H-score is calculated as (1 × weak %+2 × moderate %+3 × strongly stained %). The range of possible scores is thus 0–300.

Two cores were evaluated from each tumour. Each core was scored individually, then the mean of the two readings was calculated. Only the invasive carcinoma was assessed for staining. Noninvasive cells such as stromal cells, normal epithelial cells, benign lesions and carcinoma *in situ* were excluded from assessment. If one core was uninformative (either lost or contained no tumour tissues), the overall score applied was that of the remaining core. Previous studies have validated the use of one core to study the expression of tumour markers having heterogeneous distribution (Camp *et al*, 2000; Torhorst *et al*, 2001). One observer scored all cases, which were re-checked randomly by the same investigator after a period of time. A good correlation was found between the two estimations.

The cutoff points of expression were determined according to frequency histograms. For all markers, tumours with 5% of the neoplastic cells showing immunoreactivity were considered positive, while those with less than 5% were classified negative. Additionally, for c-ebB-3 and c-erbB-4, we considered the median as a cutoff between weak and strong expression (150 and 100, respectively). For ER, the cutoff point was taken at an H-score of 20.

### Statistical analysis

Association between the immunohistochemical findings and different clinicopathological parameters was evaluated by *χ*^2^ test. A *P*-value of <0.05 was considered to reflect a significant relationship. Survival curves were calculated by the Kaplan–Meier method. The differences between survivals were estimated by log-rank test. Multivariate Cox regression analysis was used to evaluate whether there was any independent prognostic effect of the variables on disease-free interval or overall survival.

## RESULTS

### Epidermal growth factor receptor family protein expression (Figure 1)

**Figure 1:**
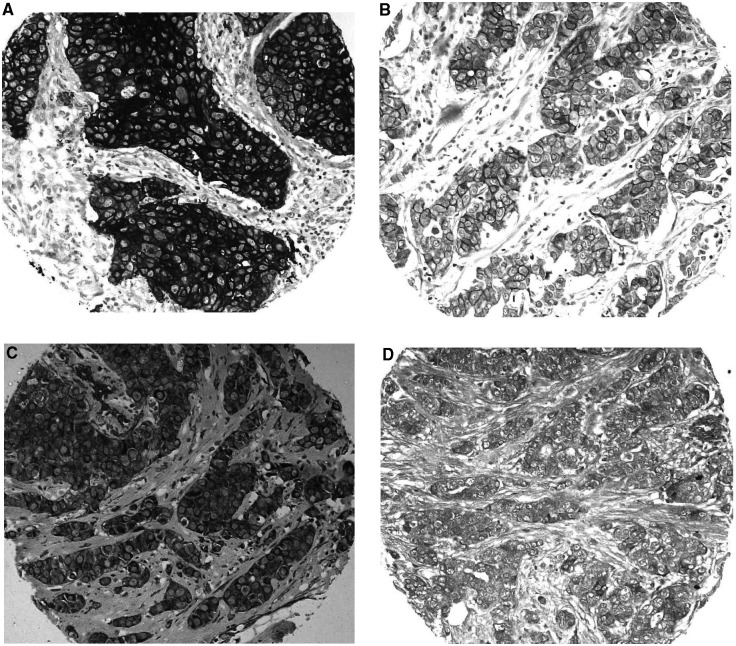
Immunohistochemical staining of breast carcinomas showing: (**A**) mixed cytoplasmic and membranous staining for EGFR, (**B**) membranous staining for c-erbB-2, (**C**) cytoplasmic staining for c-erbb-3 and (**D**) cytoplasmic staining for c-erbb-4.

Epidermal growth factor receptor protein expression was studied in 1584 cases. The expression was mainly cytoplasmic and membranous and identified in 20.1% of cases. The expression of c-erbB-2 protein was investigated in 1812 breast cancers, of which 31.8% showed membranous immunoreactivity. For the 1499 cases stained with c-erbB-3, expression was mainly cytoplasmic, with only 10.7% of cases considered negative, 44.3% weakly positive and 45% strongly positive. In the 1513 cases stained for c-erbB-4, predominantly cytoplasmic reactivity was identified in 79.3% of the tumours (34.2% weak and 45.1% strong positive), while 20.7% were negative.

Significant positive associations were found between all EGFR family members (Table 3

**Table 3:** Positive significant associations among EGFR, c-erbB-2, c-erbB-3 and c-erbB-4

**Variable**	**EGFR**	**c-erbB-2**	**c-erbB-3**	**c-erbB-4**
EGFR				
c-erbB-2	0.008			
c-erbB-3	0.001	0.013		
c-erbB-4	<0.001	0.040	<0.001	

), where 37.3% of HER1-positive tumours are HER2 positive and 24.3% of HER2-positive cases are positive for HER1 (*χ*^2^=7.01, *P*=0.008); 54.7% of HER1-positive cases are overexpressing to HER3 and 23.9% of HER3-overexpressing tumours are HER1 positive (*χ*^2^=14.54, *P*=0.001); 55.8% of HER1-positive tumours are overexpressing to HER4 and 24.3% of HER4 overexpressing cancers are positive to HER1 (*χ*^2^=17.60, *P*<0.001); 50.1% of HER2-positive tumours are overexpressing to HER3 and 34.9% of HER3-overexpressing cases are HER2 positive (*χ*^2^=8.66, *P*=0.013); 49.5% of HER2-positive tumours are overexpressing to HER4 and 34.6% of HER4-overexpressing tumours are positive to HER2 (*χ*^2^=6.41, *P*=0.040); 64.9% of HER3-overexpressing tumours are also overexpressing for HER4 and 63.7% of HER4-overexpressing tumours are also overexpressing for HER3 (*χ*^2^=351.90, *P*<0.001).

### Association between EGFR family members and different clinicopathological parameters

Table 4

**Table 4:** EGFR family members expression in relation to clinicopathological features

	**EGFR**			**ErbB2**			**ErbB3**				**ErbB4**			
**Variable**	***N***	***P***	***P*-value**	***N***	***P***	***P*-value**	***N***	**Weak**	**Strong**	***P*-value**	***N***	**Weak**	**Strong**	***P*-value**
*Grade*														
1	248	29		242	86		51	111	101		82	74	106	
2	459	59		429	173		69	226	193		138	166	194	
V3	552	230	<0.0001	562	316	0.001	39	327	375	<0.0001	92	276	378	<0.0001
Total	1259	318		1233	575		159	664	669		312	516	678	
														
*Size*														
⩽1.5 cm	449	88		450	188		58	219	234		113	184	213	
>1.5 cm	810	230	0.007	785	386	0.127	101	446	435	0.6	199	332	466	0.183
Total	1259	318		1235	574		159	665	669		312	516	679	
														
*LN stage*														
1	791	195		794	342		115	403	406		207	339	391	
2	367	94		343	178		31	202	215		83	136	233	
3	98	29	0.717	97	51	0.19	12	60	45	0.015	21	41	54	0.015
Total	1256	318		1234	571		158	665	666		311	516	678	
														
*NPI*														
Good	448	57		440	151		79	204	193		135	159	182	
Moderate	616	193		598	238		67	349	351		141	279	361	
Poor	190	68	<0.0001	194	91	<0.0001	12	111	121	<0.0001	35	78	133	<0.0001
Total	1254	318		1232	570		158	664	665		311	516	676	
														
*LR*														
Yes	89	24		77	55		18	39	39		29	43	31	
No	1153	291	0.782	1137	519	0.014	142	614	620	0.037	283	470	630	0.009
Total	1242	315		1214	574		160	653	659		312	513	661	
														
*RR*														
Yes	67	19		60	55		11	40	31		19	31	33	
No	1175	295	0.658	1154	519	<0.0001	149	613	628	0.398	293	482	628	0.672
Total	1242	315		1214	574		160	653	659		213	513	661	
														
*VI*														
Yes	395	109		370	185		37	211	222		76	162	235	
No	854	207	0.33	856	380	0.276	120	448	442	0.05	231	350	440	0.007
Total	1249			1226	565		157	659	664		307	512	675	
														
*DM*														
Yes	123	47		112	87		19	70	70		34	60	65	
No	1118	267	0.01	1100	487	<0.0001	140	583	589	0.885	277	453	596	0.586
Total	1241	314		1212	574		159	653	659		311	513	661	
														
*Death*														
Yes	106	42		93	80		15	64	60		31	54	54	
No	1136	273	0.009	1121	494	<0.0001	145	589	599	0.911	281	459	607	0.359
Total	1242	315		1214	574		160	653	659		312	513	661	
														
*ER status*														
Negative	304	164		335	208		45	202	202		59	182	206	
Positive	942	153	<0.001	865	358	<0.001	110	459	466	0.933	247	330	470	<0.001
Total	1246	317		1200	566		155	661	668		306	512	676	

shows the results of statistical analyses of the correlations between different members and clinical and pathological variables. Epidermal growth factor receptor expression was significantly associated with higher tumour grade, increasing size, higher NPI, the development of distant metastases and the incidence of death, but inversely correlated with ER status. Regarding c-erbB-2, immunoreactivity was significantly associated with poorer grade, higher NPI, local and regional recurrence, distant metastases and death, while inversely correlated to ER status. The expression of c-erbB-3 showed a significant inverse association with local recurrence, although it was significantly associated with poorer grade, lymph node disease, higher NPI and the presence of vascular invasion. The c-erbB-4 receptor expression showed a significant paradoxical association with ER status and local recurrence, while being significantly associated with poorer histological grade, higher lymph node stage, NPI and the presence of vascular invasion.

**Figure 2:**
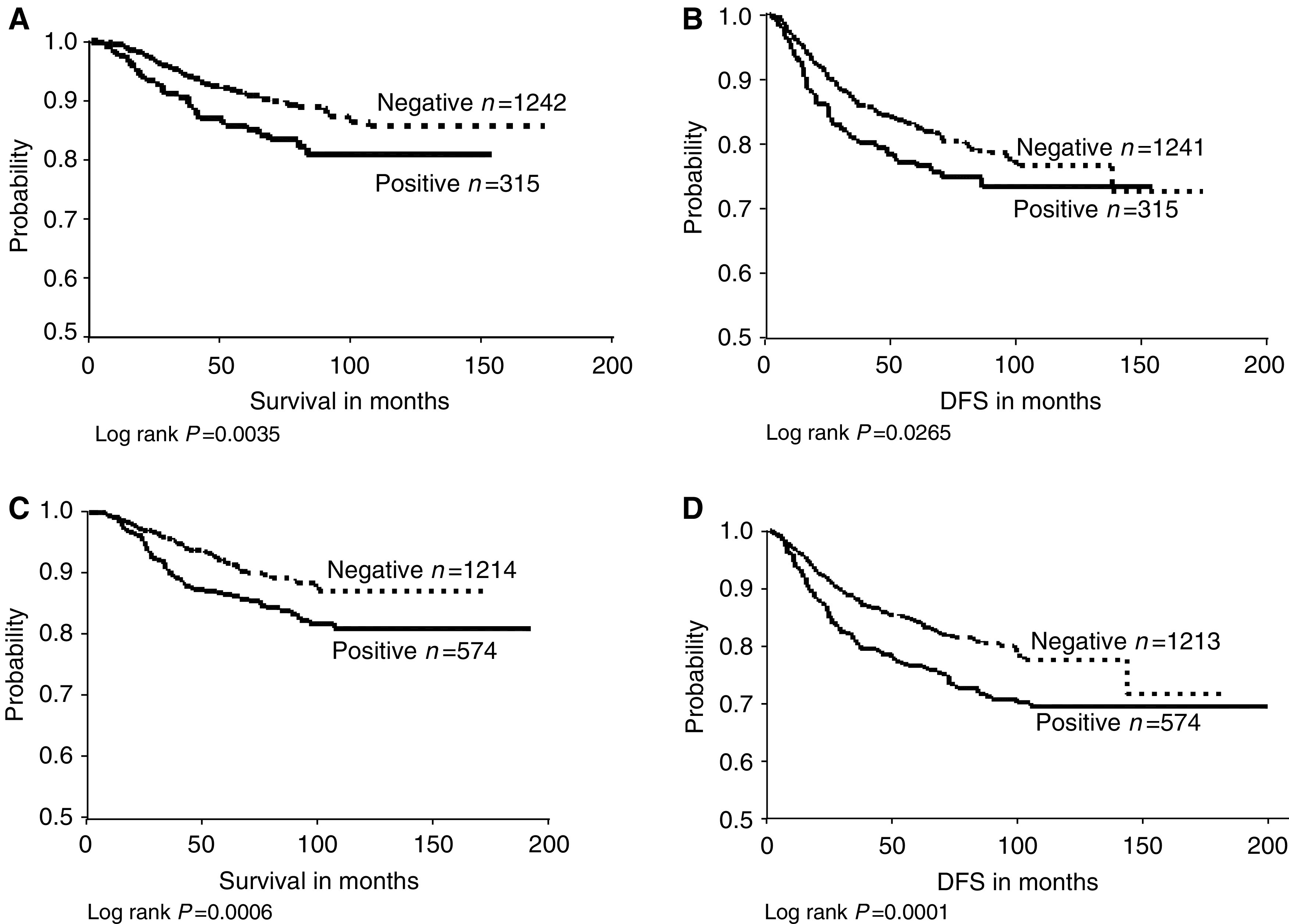
(**A**) Epidermal growth factor receptor expression in relation to overall survival demonstrating survival differences between negative and positive cases. (**B**) Epidermal growth factor receptor expression in relation to DFS showing longer DFS in negative patients. (**C**) c-erbB-2 in relation to overall survival demonstrating survival differences between negative and positive cases. (**D**) c-erbB-2 in relation to DFS with better DFS in c-erbB-2-negative cases.

Survival analyses were performed comparing the expression of the four markers in relation to both DFS and OS. We found that EGFR expression was significantly associated with shorter disease-free survival (DFS; *P*=0.0265) and overall survival (*P*=0.0035). c-erbB-2 was also significantly correlated with poorer overall survival (*P*=0.0006) and DFS (*P*=0.0001) (Figure 2A and B

). Neither c-erbB-3 nor c-erbB-4 showed a significant association with DFS (*P*=0.3401 and 0.2555, respectively) or overall survival (*P*=0.7462 and 0.7747, respectively).

Cox multivariate analyses showed that c-erbB-2 was not only significantly related to DFS and overall survival, but also that its predictive power was independent of histological grade, tumour size, lymph node status and ER status in all patients as well as patients with nodal spread. In node-negative patients, it had no prognostic power in predicting OS, while its prognostic impact is preserved in predicting DFS (Tables 5

**Table 5:** Cox multivariate regression analyses of variables in relation to disease-free survival (DFS) and overall survival (OS) in all patients

	**DFS**	**Relative risk (95% CI)**	**OS**	**Relative risk (95 % CI)**
	***P* value**		***P* value**	
*Grade*	0.004		0.152	
2 *vs* 1	0.23	1.353 (0.823–2.216)	0.203	1.995 (0.693–5.774)
3 *vs* 1	0.002	2.923 (1.462–5.844)	0.064	3.194 (0.935–10.908)
				
*LN stage*	<0.001		<0.001	
2 *vs* 1	0.879	1.035 (0.667–1.604)	0.592	0.837 (0.433–1.607)
3 *vs* 1	<0.001	3.143 (1.689–5.848)	<0.030	2.549 (1.097–5.923)
				
*Size*				
⩽1.5 *vs* >1.5	<0.034	1.449 (1.028–2.044)	<0.062	1.688 (0.975–2.924)
				
*NPI*	0.024		0.13	
Moderate *vs* good	0.032	0.520 (0.285–0.947)	0.483	1.410 (0.539–3.636)
Poor *vs* good	0.343	0.633 (0.243–1.629)	0.125	3.003 (0.773–12.218)
				
*ER*				
Positive *vs* negative	0.004	0.651 (0.483–0.871)	<0.001	0.492 (0.334–0.725)
				
*EGFR*				
Positive *vs* negative	0.661	1.074 (0.780–1.479)	0.271	1.257 (0.837-1.888)
				
*c-erbB-2*				
Positive *vs* negative	0.005	1.455 (1.121–1.887)	0.009	1.605 (1.125–2.289)
				
*c-erbB-3*	0.22		0.914	
Weak *vs* negative	0.082	0.690 (0.454–1.048)	0.786	0.916 (0.483–1.723)
Strong *vs* negative	0.116	0.721 (0.454–1.145)	0.971	0.987 (0.494–1.972)
				
*c-erbB-4*	0.045		0.255	
Weak *vs* negative	0.241	0.808 (0.566–1.154)	0.353	0.786 (0.473–1.306)
Strong *vs* negative	0.015	0.616 (0.416–0.912)	0.104	0.630 (0.361–1.099)

and 6

**Table 6:** Cox multivariate regression analyses of variables in relation to overall survival and disease-free survival in patients with and without nodal spread

	**DFS**	**OS**
	***P*-value**	***P* value**
*LN-positive patients*		
Grade	0.050	0.120
LN stage		
3 *vs* 2	<0.001	<0.001
Size	0.044	0.910
NPI	0.862	0.241
ER	0.009	0.001
EGFR	0.080	0.143
c-erbB-2	0.056	0.014
c-erbB-3	0.195	0.133
c-erbB-4	0.113	0.051
		
*LN-negative patients*		
Grade	0.064	0.605
Size	0.214	0.033
NPI	0.133	0.298
ER		
Positive *vs* negative		
0.169		
0.133		
EGFR	0.343	0.947
c-erbB-2	0.031	0.144
c-erbB-3	0.395	0.432
c-erbB-4	0.357	0.954

).

### Co-expression of EGFR family members and their association with OS and DFS

To define the frequency of dimers formation, we combined the expression of the four markers in the 1406 cases of breast cancer in which the results were available for all four members of the EGFR family. In this part of the analysis, c-erbB-3- and c-erbB-4-overexpressing cases were considered to be those showing immunohistochemical positivity above the median level, while a level below the median was considered as normal expression.

The frequencies and percentage of different dimers are shown in Table 7

**Table 7:** Frequencies of hetero- and homodimer formation among different family members

**Receptor expression**	**No.**	**%**
EGFR/c-erbB-2	25	1.8
EGFR/c-erbB-3	28	2
EGFR/c-erbB-4	33	2.3
c-erbB-2/c-erbB-3	51	3.6
c-erbB-2/c-erbB-4	47	3.3
c-erbB-3/c-erbB-4	192	13.7
EGFR/c-erbB-2/c-erbB-3	17	1.2
EGFR/c-erbB-2/c-erbB-4	14	1
EGFR/c-erbB-3/c-erbB-4	63	4.5
c-erbB-2/c-erbB-3/c-erbB-4	108	7.7
EGFR/c-erbB-2/c-erbB-3/c-erbB-4	42	3
EGFR/EGFR	51	3.6
c-erbB-2/c-erbB-2	132	9.4
c-erbB-3/c-erbB-3	126	9
c-erbB-4/c-erbB-4	139	9.9
All negative	338	24
Total	1406	100

.

The Kaplan–Meier estimates (Table 8

**Table 8:** Kaplan–Meier estimates of the associations between the expression of heterodimers and homodimers in relation to overall survival and disease free survival

		**OS**		**DFS**
**Receptor co-expression**	**No.**	***P*-value**	**No.**	***P*-value**
EGFR/c-erbB-2	25		25	
Other cases	1359	0.7109	1358	0.8442
EGFR/c-erbB-3	28		28	
Other cases	1356	0.2998	1355	0.7583
EGFR/c-erbB-4	33		33	
Other cases	1351	0.1508	1350	0.7349
c-erbB-2/c-erbB-3	51		51	
Other cases	1333	0.0354	1332	0.0423
c-erbB-2/c-erbB-4	46		46	
Other cases	1338	0.7822	1337	0.7968
c-erbB-3/c-erbB-4	187		186	
Other cases	1197	0.0114	1197	0.0634
EGFR/c-erbB-2/c-erbB-3	17		17	
Other cases	1367	0.2825	1366	0.5716
EGFR/c-erbB-2/c-erbB-4	14		14	
Other cases	1370	0.0024	1369	0.0054
EGFR/c-erbB-3/c-erbB-4	60		60	
Other cases	1324	0.5969	1323	0.752
c-erbB-2/c-erbB-3/c-erbB-4	107		107	
Other cases	1277	0.736	1276	0.6872
EGFR/c-erbB-2/c-erbB-3/c-erbB-4	42		42	
Other cases	1342	0.0985	1341	0.4445
EGFR/EGFR	51		51	
Other cases	1333	0.5	1332	0.3559
c-erbB-2/c-erbB-2	132		132	
Other cases	1252	0.4423	1251	0.0175
c-erbB-3/c-erbB-3	125		125	
Other cases	1259	0.613	1258	0.1289
c-erbB-4/c-erbB-4	131		131	
Other cases	1253	0.4136	1252	0.0086
All negative	335		335	
Other cases	1049	0.2859	1048	0.8463

) for the groups of combined co-expression of two or more receptors and both of overall and relapse-free survival showed a significant positive correlation between combined c-erbB-2 and c-erbB-3 expression and reduced OS (Figure 3A and B

**Figure 3:**
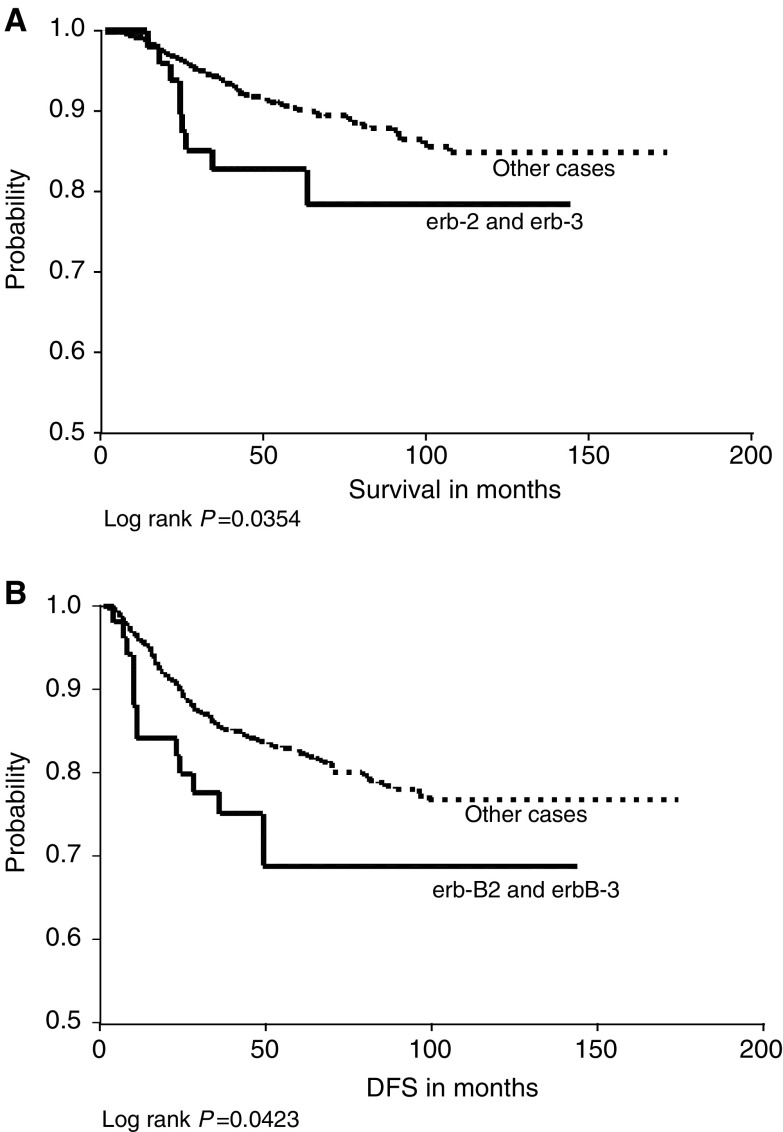
(**A**) c-erbB-2 and c-erbB-3 co-expression *vs* other cases in relation to overall survival. (**B**) c-erbB-2 and c-erbB-3 co-expression *vs* other cases in relation to DFS.

). The same relationship was also noticed with combined expression of EGFR, c-erbB-2 and c-erbB-4 in relation to OS and DFS (Figures 4A and B

**Figure 4:**
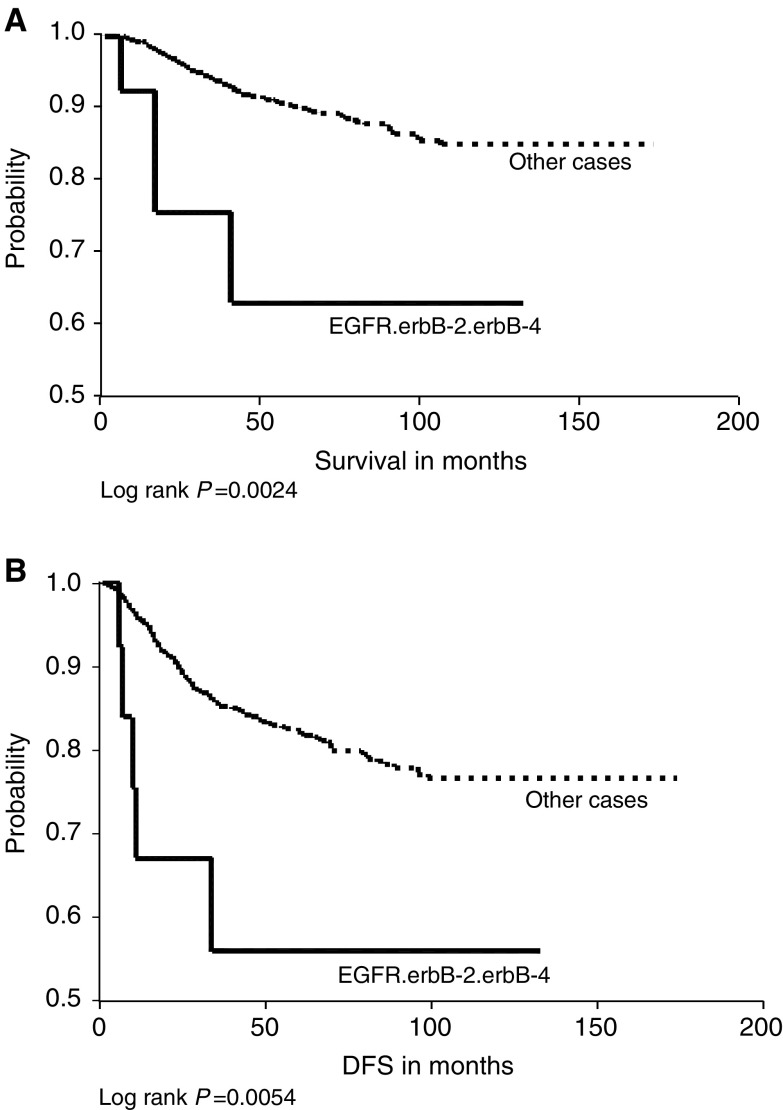
(**A**) Epidermal growth factor receptor/c-erbB-2/c-erbB-4 co-expression *vs* other cases in relation to overall survival. (**B**) Epidermal growth factor receptor, c-erbB-2 and c-erbB-4 co-expression *vs* other cases in relation to DFS.

) and for c-erbB-2 homodimers in relation to DFS (Figure 6) only. Conversely, the combined expression of c-erbB-3 and c-erbB-4 was significantly associated with a better OS and DFS (Figure 5A and B

**Figure 5:**
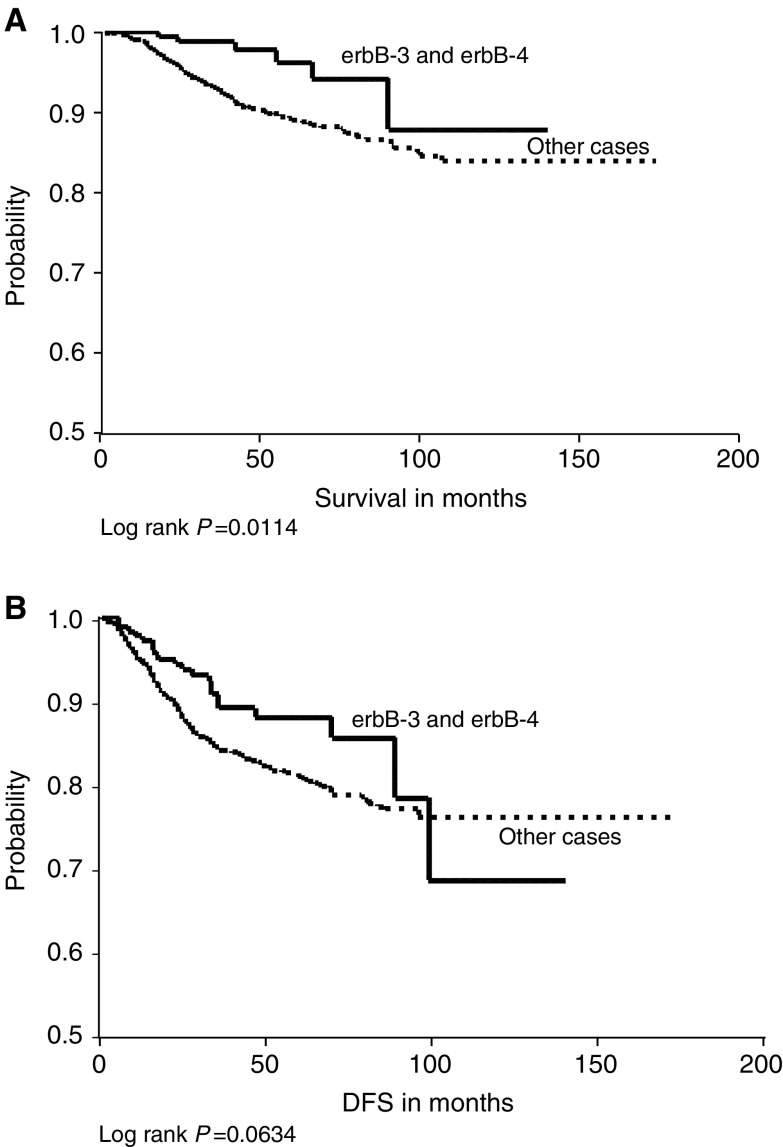
(**A**) c-erbB-3 and c-erbB-4 co-expression *vs* other cases in relation to overall survival. (**B**) c-erbB-3 and c-erbB-4 co-expression *vs* other cases in relation to DFS.

, 6

**Figure 6 fig6:**
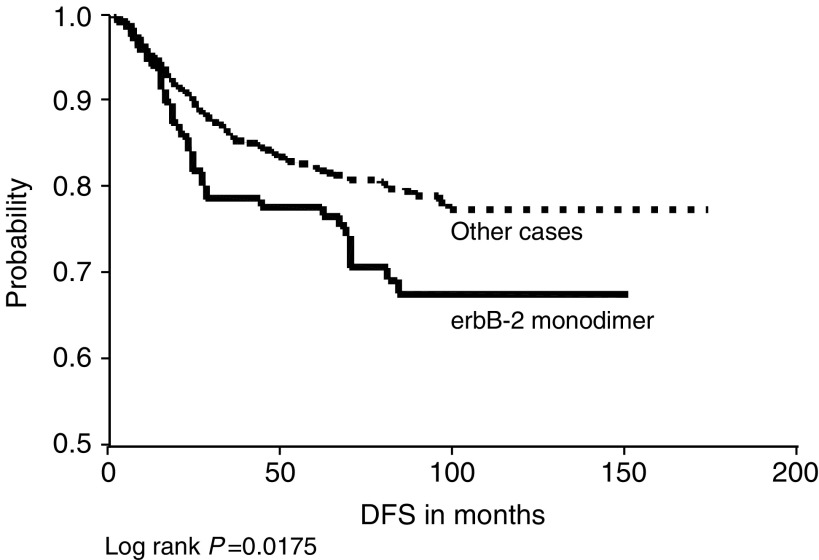
cerbB-2 homodimer expression *vs* other cases in relation to DFS.

). Also, c-erbB-4 homodimer was significantly associated with a better DFS (Figure 7

**Figure 7:**
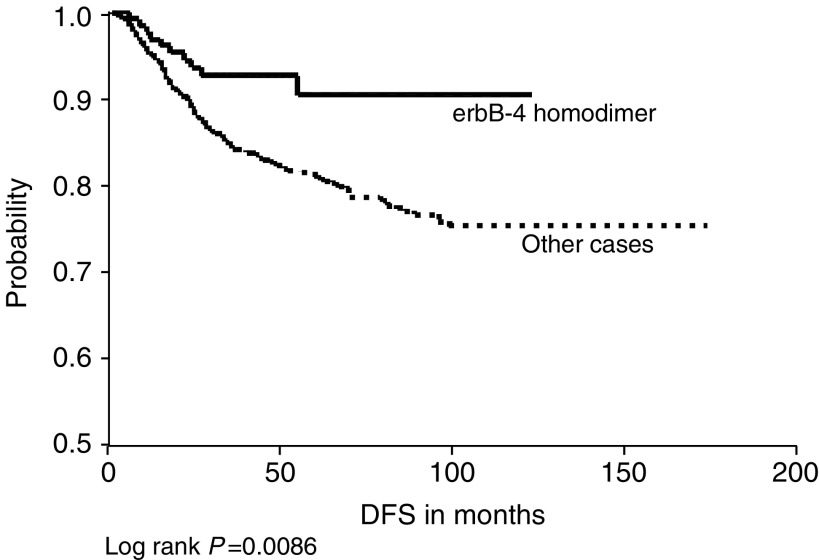
cerbB-4 homodimer expression *vs* other cases in relation to DFS.

).

**Figure 8:**
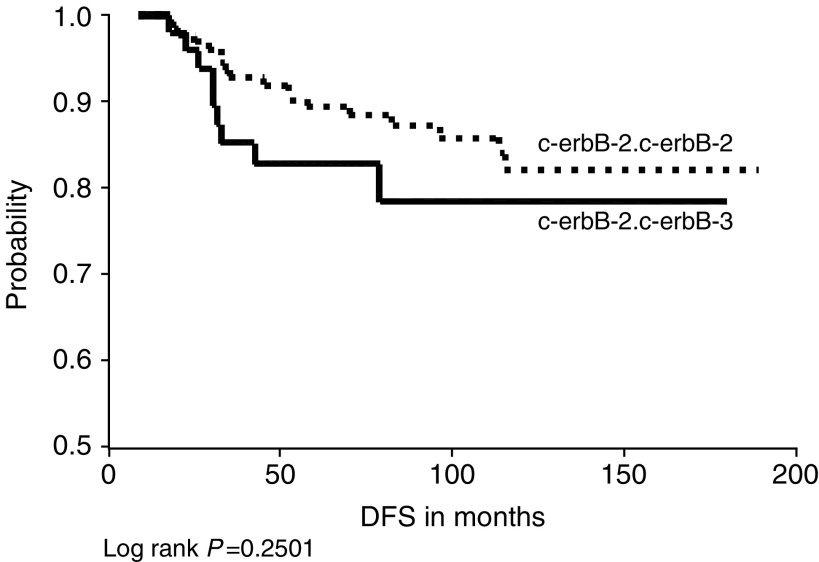
c-erbB-2/c-erbB-2 homodimer *vs* c-erbB-2/c-erbB-3 heterodimer expression in relation to overall survival.

We were interested to see whether there was a significant difference between c-erbB-2/c-erbB-3 heterodimer expression and c-erbB-2/c-erbB-2 homodimer expression in relation to DSF and OS. Although no significant differences were detected between these two subgroups in relation to DFS (*P*=0.6606) and OS (*P*=0.2501), we found that OS was worse in patients with tumours expressing c-erbB-2/c-erbB-3 heterodimers compared to those with tumours expressing c-erbB-2/c-erbB-2 homodimer (Figure 8

).

## DISCUSSION

In this study, expression of EGFR was identified in 20.1% of cases, consistent with previous studies where EGFR expression has been reported in 14–65% of breast cancer (Suo *et al*, 1998; Walker and Dearing, 1999). c-erbB-2 overexpression was detected in 31.8%, in line with previous reports showing 10–34% expression (Pauletti *et al*, 1996; Ross and Fletcher, 1999). Expression of c-erbB-3 and c-erbB-4 was 89.3% (45% strongly positive) and 79.3% (45.1% strongly positive), respectively. These findings are also in accordance with previous work which has identified c-erbB-3 expression in 65–95% of cases and c-erbB-4 in 58–82% of breast cancers (Travis *et al*, 1996; Naidu *et al*, 1998; Kew *et al*, 2000; Suo *et al*, 2002; Witton *et al*, 2003).

In this study, EGFR expression was significantly associated with features recognised to reflect poor prognosis, including high (poor) histological grade, high NPI score, negative ER status, larger tumour size, the development of distant metastases and death. Previous studies have reported the same relationships with tumour grade (Suo *et al*, 1998), negative ER status and reduced survival (Toi *et al*, 1990). Other studies have failed to find an association with grade, tumour size and lymph node status (Lewis *et al*, 1990).

We have confirmed the previously reported significant correlation identified between c-erbB-2-expressing tumours and poorer tumour grade, ER negativity (Lovekin *et al*, 1991; Suo *et al*, 2002; Witton *et al*, 2003; Zhang *et al*, 2003), high NPI score, local and regional recurrence, distant metastases and death from breast cancer. Our finding regarding the positive association between the development of distant metastatic disease and c-erbB-2 expression corroborates the findings of Tan *et al* (1997), who transfected c-erbB-2 gene in the very low c-erbB-2-expressing MDA-MB-435 human breast cancer cell line and found that its overexpression enhanced the metastatic potential of cancer cells.

A significant association between c-erbB-2 overexpression and poor outcome in the form of short OS and DFS was found, as previously observed in several series (Lovekin *et al*, 1991; Winstanley *et al*, 1991; Witton *et al*, 2003). Winstanley *et al* (1991) showed the independent prognostic effect in relation to OS. We have also demonstrated that c-erbB-2 expression independently predicted for poor OS and DFS in the whole population of breast cancer patients as well as in those with node-positive disease. The significant impact of c-erbB-2 overexpression/amplification on poor outcome in patients with positive nodal metastases is well established (Lovekin *et al*, 1991; O'Reilly *et al*, 1991).

In this series, c-erbB-3 expression was positively associated with grade, lymph node metastases, poor NPI and vascular invasion, while it was inversely associated with local recurrence. No relation was identified with either ER status or survival. Previous reports have recorded an association between c-erbB-3 overexpression and poor prognostic features such as high grade (Naidu *et al*, 1998) and lymph node spread (Lemoine *et al*, 1992). Some other studies have previously noted an association with ER status, which we did not find in the present large series. For example, Knowlden *et al* (1998) reported a significant association with positive ER status and good response to endocrine therapy for cases overexpressing c-erbB-3, while others have failed to find associations with ER status (Travis *et al*, 1996; Naidu *et al*, 1998). We have also, in the present study, identified an inverse relation with local recurrence, while the reverse was reported by Travis *et al* (1996), where they found that moderate to strong expressing tumours were more likely to develop local recurrence compared with weak expressing and negative tumours (Travis *et al*, 1996). The discrepancies in such relations may be partly explained by the discovery of a c-erbB-3-soluble form, p85-soluble ErbB-3 (sErbB-3), a potent negative regulator to heregulin, which inhibits heregulin-induced phosphorylation of c-erbB-2, c-erbB-3 and c-erbB-4 by competing for heregulin binding (Lee *et al*, 2001), although other potential explanations may also be variations in antibodies and cutoffs used.

Very little is known with respect to the prognostic significance of c-erbB-4 in breast cancer. In this study, we have demonstrated that its overexpression was associated with higher grade, nodal metastases, poor NPI and vascular invasion, and inversely correlated with ER status and local recurrence. No significant association was identified in relation to survival. Most studies presented to date do not have congruent findings regarding the prognostic effect of c-erbB-4 in breast cancer. Some studies linked c-erbB4 overexpression with a well-differentiated phenotype (Kew *et al*, 2000; Suo *et al*, 2002), positive ER status (Knowlden *et al*, 1998; Suo *et al*, 2001) and longer survival (Suo *et al*, 2002; Witton *et al*, 2003). Another study associated overexpression with poor survival in lymph node-positive cases (Lodge *et al*, 2003). The significant inverse association between c-erbB-4 expression and ER status observed in the present study has only been recorded in one study by Vogt *et al* (1998), who demonstrated an inverse association between c-erbB-4 gene amplification and ER status in breast cancer (Vogt *et al*, 1998). These conflicting results may be related to different scoring systems used, different definitions of overexpression, the use of different antibodies, antigen retrieval techniques and the heterogeneous populations of patients selected for the studies. Variable responses may also be evoked by signals generated from either c-erbB-4 homodimers or heterodimers with other members, the level of which depends upon the context of expression of other receptors. This may explain the association of c-erbB-4 overexpression with some poor prognostic features in our study, as a great many of cases co-expressed one or more other receptors along with c-erbB-4. In support of this concept, a poor prognostic association has been reported in relation to the co-expression of c-erbB-2 and c-erbB-4 in childhood medulloblastoma (Gilbertson *et al*, 1997). Another important factor in relation to the diverse behaviour of c-erbB-4 is the type of isoform expressed by the tumour. For example, the CYT-1 isoform mediates proliferation as well as chemotaxis and survival signals, whereas the CYT-2 isoform stimulates proliferation and growth only (Junttila *et al*, 2000).

The hallmark of this family of growth factor receptors is the ability of its members to act or function synergistically with another receptor through dimerisation. ErbB-2 represents the preferred heterodimerisation partner of all other receptors of the family, and the preferred dimerisation partner of ErbB-2 is ErbB-3 (Tzahar *et al*, 1996). We found that 50.1% of c-erbB-2-expressing tumours also overexpressed c-erbB-3 and that this heterodimerisation was significantly associated with poor OS and DFS. This dimer, formed of ligand-deficient c-erbB-2 and kinase-deficient c-erbB-3, is known to form the most potent signalling pair in terms of growth and transformation (Alimandi *et al*, 1995; Wallasch *et al*, 1995). Therefore, the detection of the co-expression of these two receptors may have more clinical and prognostic significance than the detection of expression of each receptor separately.

Although there was a significant association between EGFR and c-erbB-2 expression in this study, dimers formed of these receptors alone are less frequent than other dimers; however, their combined expression was more frequently common with c-erbB-3 and c-erbB-4. Our study was one of few studies that addressed the expression of the four receptors together compared to previous studies that considered the expression of EGFR and c-erbB-2 ignoring the other receptors (Tsutsui *et al*, 2003).

In spite of the fact that the immunohistochemical expression of both of EGFR and c-erbB-2 are linked with poor survival, heterodimers formed of this pair had no significant association with survival. It was surprising that EGFR and c-erbB-2 alone had prognostic impacts whereas the combination of both was of no significance, which disagree with literature (Toi *et al*, 1994; Suo *et al*, 2002). We have no explanation for this contradiction, but there are several factors that potentially contribute to these inconsistent findings, including the use of different antibodies and different cutoffs and definition of overexpression. Changing the antibodies against EGFR and/or c-erbB-2 might overcome this contradiction.

Cases co-expressing more than two family members, the combination of EGFR, c-erbB-2 and c-erbB-4 had a highly significant worse OS and DFS compared to other cases. The co-expression of EGFR and c-erbB-2 has an additive adverse effect in relation to survival (Toi *et al*, 1994; Suo *et al*, 2002).

Another interesting heterodimer identified in our study is that of c-erbB-3 and c-erbB-4; cases which expressed both receptors had a significantly better OS and DFS (trend) compared with other dimers. We have no explanation for this finding; however, cell line studies have shown that NDF induced mitogenesis in cells expressing c-erbB-3 or c-erbB-4 but not transformation, which was induced only when either EGFR or c-erbB-2 were coexpressed with c-erbB-3 or c-erbB-4 (Zhang *et al*, 1996). This suggests that c-erbB-3 and c-erbB-4 may activate signalling pathways that are different from those activated by EGFR and c-erbB-2, or an additional pathway may be needed to induce transformation.

In the present study, c-erbB-2 homodimers were significantly associated with a poorer DFS. In spite of being a ligandless receptor, cell line studies have shown that its overexpression induced homodimerisation that was sufficient to induce growth, malignant transformation (Di Fiore *et al*, 1987; Brennan *et al*, 2000) and cell migration (Verbeek *et al*, 1998). Conversely, we found that c-erbB-4 homodimers were significantly associated with a better DFS compared with others. A previous study has similarly recorded an association with a differentiated phenotype and with better prognosis in breast cancer (Suo *et al*, 2002), and another recent study has reported that breast cancer cases overexpressing c-erbB-4 only were the best of all cases regarding outcome (Witton *et al*, 2003).

The diversity identified in our study, in terms of survival, between different heterodimers and homodimers may be useful in subgrouping of breast cancer patients with significantly differing outcome. These data suggest that the predictive value of EGFR family overexpression may be optimised by combining information about the expression of all of the family members, rather than the assessment of a single receptor in isolation.

There are other essential components of the EGFR family network, which have not been studied here; 10 or more ligands and the proteins that are involved in EGFR family-induced pathways. The receptors are conduits for the ligand-activated signalling pathways and, although their expression levels clearly do contain useful information, the precision of the analysis can only be improved in the future by using antibodies for detection of such proteins.
